# Microbiota Manipulation by Probiotics Administration as Emerging Tool in Cancer Prevention and Therapy

**DOI:** 10.3389/fonc.2020.00679

**Published:** 2020-05-22

**Authors:** Concetta Panebianco, Tiziana Latiano, Valerio Pazienza

**Affiliations:** ^1^Division of Gastroenterology, Fondazione IRCCS Casa Sollievo della Sofferenza, San Giovanni Rotondo, Italy; ^2^Oncology Department, Fondazione IRCCS Casa Sollievo della Sofferenza, San Giovanni Rotondo, Italy

**Keywords:** microbiota, prebiotic, animal model, cancer, cancer therapies

## Abstract

A growing body of literature indicates that microbiota plays a significant role in the development and curability of cancer, essentially due to the microbial ability to modulate immune and inflammatory responses to cancer and therapeutic treatments. Probiotics consumption, either in the form of food or supplements, is an easy and feasible way to manipulate microbiota composition and a number of recent researches have shown that it may represent a valid approach to prevent cancer onset and progression, to improve the clinical efficacy of the current anticancer treatments, and to mitigate the harmful adverse events of chemo- and radiotherapy, which often lead to scale drug doses, to delay or interrupt treatments. In this review, we gather the main *in vivo* studies on the current topic, focusing on the beneficial effects and underlying mechanisms provided by bacterial and yeast probiotics and their combination, in the setting of various types of cancers and different therapeutic protocols. These findings will likely open the way to consider, in future, regular probiotics intake as an adjuvant strategy in cancer prevention and management.

## Introduction

Cancer is a global health burden, representing the second leading cause of death all over the world. According to the World Health Organization (WHO), it is estimated that about one-third of deaths from cancer are caused by modern behavioral and dietary habits, including low fruit and vegetable consumption, sedentary lifestyle, obesity, smoking, and alcohol intake^1^. Among these modifiable risk factors, a growing interest in diet impact on health and disease has been fueled by the blooming of microbiota studies following the disclosure of the so-called “our other genome,” a gene catalog of the human gut microbiome (1). It has been largely demonstrated that gut microbiota has a key role in maintaining health status and that food is one of the main tools to shape its composition (2, 3). The impact of different dietary patterns and food constituents on microbiota composition has been exhaustively reviewed elsewhere (2, 4, 5). Particular relevance to human health is given to the intake of fruits and vegetables as a source of indigestible fibers, which are known to benefit the host gut environment by selectively promoting the growth of beneficial bacteria, known as “probiotics.” The term “probiotic” comes from the Latin preposition “pro,” which means “for, in favor of” and the Greek word “bios” meaning “life” and, according to the definition by the WHO, refers to “live microorganisms, which, when administered in adequate amounts, confer a health benefit on the host” (6). Probiotics can be introduced in the form of food (such as yogurt, kefir, milk products, and fermented foods) or as supplements, and arrive alive in the intestine. The mechanisms through which these beneficial microbes influence host health include synthesis of vitamins and essential amino acids, production of metabolites such as short chain fatty acids derived from fibers' fermentation, reinforcement of the intestinal mucosal barrier, defense against pathogen colonization, detoxification of carcinogenic compounds, and stimulation of the host immune system (2, 7). Most probiotics belong to the genera *Bifidobacterium* and *Lactobacillus*, indigenous inhabitants of the human and animal gastrointestinal tract. *Lactobacillus* and other less common probiotics, such as *Lactococcus, Enterococcus*, and *Streptococcus*, are lactic acid bacteria, which convert sugars into lactic acid thus lowering the intestinal pH and hindering potentially pathogenic bacteria (8). Moreover, in addition to bacteria, yeast strains belonging to the genus *Saccharomyces* are also numbered among the probiotics (8).

Several studies have been carried out both *in vitro* and *in vivo* claiming the efficacy of probiotics in the prevention and treatment of cancer; however, since the real effect of probiotics can be evaluated only in *in vivo* studies (9), this review will summarize the main findings in human and animal studies concerning the ability of probiotics to affect cancer development, enhance effectiveness, or limit toxicity of the conventional anticancer treatments.

## Probiotics in Cancer Prevention and Progression

One of the best known and most investigated probiotic strains is *Lactobacillus casei* Shirota (LcS), a microorganism providing many health-favorable effects, mainly boosting the host immune system and preventing infectious gut diseases (10).

Several *in vivo* studies have evaluated the protective effects of LcS against different types of cancer (11–14). In a rat model of azoxymethane (AOM)-induced colon cancer, administration of a diet containing LcS significantly decreased carcinogenesis in terms of tumor incidence and multiplicity, likely by increasing the number of cytotoxic T lymphocytes (11). A later study on Japanese patients with a previous history of at least two colorectal cancers, showed that regular consumption of LcS, though not affecting the occurrence of new tumors, significantly reduced moderate and severe atypia (14). A case-control study carried out on Japanese women highlighted that regular intake of LcS in combination with isoflavones from soy since adolescence was protective against the risk of breast cancer (12). Replicating this experimental design in a rat model of chemically-induced breast cancer demonstrated that LcS consumption (alone and in combination with soymilk) decreased mammary tumor volumes (13). Also, *Lactobacillus plantarum* LS/07 was shown to slow mammary tumor growth in rats injured with 7,12-dimethylbenz[a]anthracene. Daily administration of this strain before and after cancer induction significantly decreased tumor frequency, increased CD4^+^ and CD8^+^ T cells in tumor tissues, and decreased TNFα in the serum (15). Evidence of a cancer protective action was also provided for another strain of *L. casei*, namely *L. casei* BL23, which prevented colorectal cancer development in two different mouse models induced by 1,2-dimethylhydrazine (DMH) and AOM, respectively (16, 17). In the first case, *L. casei* BL23 was found to modulate host immunity, shifting toward a Th17-response (16), whereas in the second case inhibition of cell proliferation and promotion of apoptosis by the probiotic were demonstrated (17). A breast cancer-protective action was demonstrated for *Lactobacillus acidophilus* ATCC4356 strain which, administered to mice before and after tumor implantation, significantly slowed its growth, increased the production of the T cell-stimulating IL-12, while decreasing that of the immunosuppressive TGF-β (18). *L. acidophilus*, combined with *Bifidobacterium bifidum* and *Bifidobacterium infantum* also proved to be effective in reducing tumor incidence, multiplicity, and volume in a rat model of DMH-induced colon cancer. Daily intake of this probiotic cocktail decreased the abundance of potentially pathogenic gut bacteria, reduced intestinal inflammation, and promoted the intestinal epithelial barrier function by enhancing TLR-2 signaling (19). Indeed, TLR-2 is a receptor for bacterial molecules whose stimulation maintains the integrity of intestinal tight junctions against injuries and whose deficiency disrupts epithelial barrier function, thus worsening colonic inflammation (20). Further, TLR-2 promotes colon cancerogenesis (21). Consistently, Kuugbee et al. (19) observed that increased expression of TLR2 upon probiotics administration was accompanied by higher levels of the junction proteins ZO-1 and occludin. Similar results were obtained by the same group when DMH-injured rats were administered daily with *Lactobacillus rhamnosus* GG: colon tumor incidence, multiplicity, and volume significantly decreased, in addition to intestinal inflammation (22). Again, in the setting of DMH-induced colon carcinogenesis, the probiotics *Bacillus subtilis* and *Clostridium butyricum* separately administered to cancer-induced mice significantly decreased tumor incidence and size, compared to injured mice not receiving probiotics. Inhibition of carcinogenesis was accompanied by a decreased number of Th2 and Th17 lymphocytes in the spleen and an increased number of CD4^+^ and CD8^+^ T cells in the peripheral blood of mice receiving *B. subtilis* or *C. butyricum*, compared to animals not administered with probiotics (23). Moreover, *C. butyricum* ATCC 19398 strain was tested against high fat diet-induced tumorigenesis in APC^Min^ mice, genetically predisposed to intestinal neoplasms. Animals treated with this probiotic developed significantly less intestinal cancers, likely by modulation of gut microbiota and suppression of the Wnt/β-catenin signaling (24). The latter is a pathway aberrantly activated in colorectal cancer, in which Wnt stimulation suppresses the β-catenin degradation system, resulting in its accumulation and nuclear translocation with consequent expression of target genes driving cell proliferation (25).

The commercially available probiotic cocktail VSL#3 containing *Lactobacillus plantarum, Lactobacillus delbrueckii* subsp. *bulgaricus, Lactobacillus paracasei, Lactobacillus acidophilus, Bifidobacterium breve, Bifidobacterium longum, Bifidobacterium infantis*, and *Streptococcus salivarius* subsp*. Thermophilus*, known to alleviate colitis, was also shown to prevent colitis-associated adenocarcinoma in mice injured with dextran sulfate sodium (DSS). In particular, both prophylactic treatment before colitis induction and concurrent treatment with DSS led to decreased inflammation and reduced colonic dysplasia. Interestingly, even after 15 cycles of DSS, none of the mice receiving VSL#3 concurrently with DSS developed adenocarcinoma, whereas 20% of animals receiving preventive VSL#3 and 45% of mice not taking probiotics did (26). Furthermore, an Italian clinical study on human volunteers followed-up for 12 years after enrollment as concerns the development of colorectal cancer, verified an inverse association between yogurt (containing *S. thermophilus* and *L. delbrueckii* subsp*. bulgaricus*) consumption and colorectal cancer occurrence, which was more evident in men than in women (27).

In all the above-mentioned studies, the probiotic agent was represented by one or more bacterial strains, above all lactic acid bacteria; nevertheless, non-pathogenic yeasts, such as *Saccharomyces boulardii*, can also be listed in the family of probiotics (8, 28).

A study by Chen et al. demonstrated that daily *S. boulardii* administration to APC^Min^ mice significantly reduced tumor number and size and the grade of dysplasia. These effects were mediated by the inhibition of EGFR and AKT proliferative pathways (29). More recently, *S. boulardii* administration to a mouse model of colitis-associated carcinogenesis induced by AOM/DSS was shown to reduce tumor incidence and size, to decrease colonic levels of the pro-inflammatory cytokines TNF-α and IL-6, and restore a balanced microbiota (30).

## Probiotics and Anticancer Therapies' Effectiveness

In addition to cancer preventive action, some studies revealed a supportive role for probiotics toward chemotherapy, in terms of improved pharmacological response (31–33). A clinical trial on patients subjected to transurethral resection of bladder cancer treated with epirubicin showed that daily administration of LcS for 1 year significantly increased the recurrence-free survival compared to epirubicin alone, though not affecting progression-free and overall survival (31). Effectiveness of *L. acidophilus* in boosting cisplatin action was provided in a mouse model of lung cancer: oral intake of this probiotic together with chemotherapy resulted in decreased tumor volume and extended survival with respect to cisplatin alone. These results were accompanied by lower expression of the oncoprotein VEGFA, higher expression of the tumor suppressors BAX and CDKN1B, and by increased serum levels of IL-6 and IFN-γ together with reduced levels of the immunosuppressive IL-10 in *L. acidophilus*/cisplatin combined treatment versus cisplatin alone (32). The use of a commercial probiotic mixture containing *L. acidophilus, L. paracasei*, two strains of *B. lactis* and *B. bifidum* turned out to be advantageous in enhancing 5-Fluorouracil (5-FU) action in a rat model of colorectal cancer induced by DMH. Administration of probiotics together with chemotherapy attenuated the degree of tumor malignancy compared to 5-FU alone, since 40% of animals developed tubular adenoma and 60% carcinoma *in situ*, compared to 100% of carcinoma *in situ* developed by rats only receiving chemotherapy (33).

Evidence has been provided to suggest that probiotics also improve the outcome of anti-cancer immunotherapy protocols (34–36). A mixture of *B. bifidum, B. longum, B. lactis*, and *B. breve*, administered to mice after melanoma implantation concurrent with anti-PD-L1 immunotherapy resulted in a significantly decreased tumor volume and increased number of tumor-infiltrating CD8^+^ T cells compared to anti-PD-L1 therapy alone (34). Apart from the most common probiotic microorganisms cited above, other less known bacteria demonstrated beneficial effects. Administration of *Akkermansia muciniphila*, alone or in combination with *Enterococcus hirae*, restored response to anti-PD1 therapy in melanoma or sarcoma-bearing mice with depleted gut microbiota, as demonstrated by reduced tumor size. This was likely due to an enhanced immunological response against the tumor, consisting in the accumulation of CD4^+^ T cells in mesenteric and tumor draining lymph nodes and in tumor bed, and in the increased secretion of IL-12, which normally synergizes anti-PD1 therapy (36). Finally, the impact of a daily administration of the probiotic *Escherichia coli* Nissle 1917 on anti-TGF-β immunotherapy was evaluated in mouse models of hepatocellular and breast carcinoma. Markedly decreased tumor size, reduced Ki67 expression, increased apoptosis, inhibition of metastatic potential and shift from an immune-suppressive to an immune-stimulatory microenvironment were observed in combined treatment compared to immunotherapy alone (35).

Table 1 provides further details on the probiotic treatment scheme and the anticancer therapies' schedule followed in the above-mentioned studies.

**Table 1:** Effects of probiotic intake on anticancer therapies' effectiveness.

**Probiotic(s)**	**Probiotic dosage and treatment scheme**	**Therapeutic treatment scheme**	**Effect(s)**	**Reference**
*L. casei* Shirota	Daily intake of 3 × 10^10^ bacteria for 1 year, starting after transurethral resection (TUR) and the first two epirubicin instillations	30 mg doses of epirubucin soon after TUR and after 1, 3, 4, 6, 8, 10, and 12 weeks, respectively	Increased recurrence-free survival in bladder cancer patients	(31)
*L. acidophilus*	Administration of 2 × 10^8^ CFU/mL	Intraperitoneal injection of 5 mg cisplatin/kg body weight	Decreased tumor size and extended survival in mice with implanted lung cancer	(32)
*L. acidophilus* + *L. paracasei* + two strains of *B. lactis* + *B. Bifidum*	Daily administration of 1 × 10^9^ CFU for 10 weeks	A weekly dose of 15 mg of 5-FU/kg body weight for 10 weeks	Milder aggressiveness of chemically-induced colorectal tumors in rats	(33)
*B. bifidum + B. breve* + *B. longum + B. lactis*	Administrations of 1 × 10^9^ CFU 7 and 14 days after tumor implantation	Immunotherapy with 100 μg αPD-L1 mAb 7, 10, 13, and 16 days after tumor implantation	Decreased tumor volume in mice implanted with melanoma	(34)
*A. muciniphila* or *A. muciniphila + E. Hirae*	Five 10^9^ CFU/mL administrations, the first 24 h before the first injection of anti PD-1 mAb and subsequently four times on the same day as anti-PD-1 mAb therapy	Four immunotherapy administrations at 3 day intervals of 250 μg anti-PD-1 mAb	Reduced tumor size in mice with implanted melanoma or sarcoma	(36)
*E. coli* Nissle 1917	Daily administration of 1 × 10^9^ CFU for 22 (liver cancer) or 27 days (breast cancer) strarting on the day of tumor implantation	45 mg/kg of anti-TGF-β immunotherapy for 7 consecutive days	Decreased tumor size and inhibition of metastatic potential in mice implanted with hepatocellular carcinoma or breast cancer	(35)

A very recent preliminary study, however, suggests that the intake of probiotic supplements in melanoma patients lowers the response to anti-PD1 immunotherapy by 70%, likely decreasing gut microbiota diversity (37). These controversial findings, which stand in conflict with the general perception of probiotics being beneficial and with previous results, deserve further investigations.

## Probiotics and Anticancer Therapies' Toxicity

Apart from improving their clinical efficacy, probiotics also hold promise for relieving some adverse reactions associated with the conventional anticancer treatments, which often make it necessary to scale drug doses and to delay or stop therapies. Intestinal damage represents a major debilitating complication of chemo- and radiotherapies, mostly presenting in the form of diarrhea and mucositis, a painful inflammatory/ulcerative condition of the mucosa (38).

Daily *L. rhamnosus* GG supplementation in colorectal cancer patients during 5-FU-based adjuvant chemotherapy was shown to decrease severe diarrhea, abdominal discomfort, and the need to reduce chemotherapy doses with respect to patients not receiving probiotics (39). Other probiotics have also been successfully used to alleviate 5-FU-induced intestinal toxicity. Oral intake of *B. bifidum* G9-1, before and during 5-FU administration in mice significantly reduced the body weight loss and the intestinal damage, as assessed by a minor shortening of the small intestine, of the villus length and a minor destruction of the crypts. In addition, a milder inflammatory response and attenuation of dysbiosis were observed upon probiotic treatment (40). Similar results were obtained in rats injured with a single dose of 5-FU and administered with *B. infantis* starting before drug injection, in which loss of body weight, villus shortening, and diarrhea occurrence, as well as expression of inflammatory factors were alleviated compared to animals only treated with chemotherapy (41). Similarly, administration of a mix of the four probiotical strains *B. breve* DM8310, *L. acidophilus* DM8302, *L. casei* DM8121, and *S. thermophilus* DM8309 to rats concomitantly treated with 5-FU caused a milder intestinal damage, as assessed by decreased severity of histological score, increased villus length, crypt depth, and mucus layer, lower expression of inflammatory cytokines and inflammatory markers, compared to rats only receiving 5-FU (42). Furthermore, 5-FU-induced intestinal mucositis was ameliorated in a mouse study by Yeung et al. (43). Animals were injected with 5-FU and concurrently administered with saline, *L. casei* variety *rhamnosus* or a mix of *L. acidophilus* and *B. bifidum*. In both probiotic treatments, body weight loss and diarrhea were less severe, villus shortening was decreased, the crypt depth restored and the expression of pro-inflammatory cytokines suppressed (43). Another chemotherapeutic drug frequently leading to intestinal toxicity is irinotecan, administered to treat advanced colorectal cancer. A pilot study on human cancer patients receiving irinotecan reported a preventive effect against diarrhea for a commercial probiotic formula containing the following 10 bacterial strains: *B. breve* HA-129, *B. bifidum* HA-132, *B. longum* HA-135, *L. rhamnosus* HA-111, *L. acidophilus* HA-122, *L. casei* HA-108, *L. plantarum* HA-119, *S. thermophilus* HA-110, *L. brevis* HA-112, and *B. infantis* HA-116. Patients taking probiotics daily during chemotherapy had less diarrhea incidence and severity, and less enterocolitis and bloating compared to those taking placebo (44). Also, the VSL#3 probiotic mix, administered before and after chemotherapy, was tested against intestinal mucositis in rats injected with a single dose of irinotecan. Weight loss, diarrhea severity, and crypt damage were decreased, as assessed by increased epithelial proliferation and reduced apoptosis (45). Other probiotic formulations are being tested in ongoing clinical trials to evaluate their effect on intestinal damage such as the OMNi-BiOTiC® 10 AAD, whose ability to reduce grade III/IV diarrhea during the FOLFIRI-based chemotherapy in metastatic colorectal cancer patients is under investigation in a Phase 2 study (NCT03705442).

The VSL#3 formula also provided intestinal benefits in patients undergoing adjuvant radiotherapy for intestinal or cervical cancer (46). The probiotic intake along the duration of the radiation therapy schedule significantly decreased enterocolitis occurrence, incidence, and severity of diarrhea and number of daily bowel movements, and delayed the use of antidiarrheal drugs (46). Likewise, the severity grade of diarrhea and the need to take anti-diarrheal drugs in cervical cancer carriers undergoing radiotherapy concurrent with cisplatin chemotherapy were significantly decreased in those patients administered with *L. acidophilus* plus *B. bifidum-*based probiotic before and during radiotherapy (47). Similar results were observed in cervical cancer patients taking *L. acidophilus* LA-5 plus *B. animalis* subsp*. lactis* BB-12 for all the duration of the radiotherapy schedule, in which diarrhea incidence and severity were decreased together with the use of anti-diarrheal medication and the number of episodes of abdominal pain (48).

Chemo- and radiotherapy may also lead to mucositis in oropharyngeal districts, besides at the intestinal level. This event is very common in patients treated for head and neck cancers (49, 50). A placebo-controlled study reported a protective role for the probiotic *L. brevis* CD2 against this adverse effect in patients with head and neck squamous cell carcinoma treated with cisplatin-based chemotherapy and radiation therapy (51). Probiotic administration during and after the end of therapy decreased incidence and severity of oral mucositis and allowed a higher number of patients to complete the anticancer treatment compared to the placebo group (51). Another recent paper investigating the effect of the same probiotic, administered according to the same scheme, however, failed to demonstrate its preventive effect on chemoradiotherapy-induced oropharyngeal mucositis in head and neck cancer patients (50). On the other hand, another study demonstrated that head and neck cancer carriers undergoing cisplatin chemotherapy and radiotherapy and taking a probiotic mix including *B. longum, L. lactis*, and *Enterococcus faecium* all along the treatment developed less severe oral mucositis compared to the placebo group. This benefit was achieved by enhancing patients' immunity, since probiotics significantly counteracted the reduction of CD8^+^, CD4^+^, and CD3^+^ T cells induced by the anticancer treatments (49).

More details on the probiotic treatment scheme and the anticancer therapies' schedule adopted in these studies are provided in Table 2.

**Table 2:** Effects of probiotic intake on anticancer therapies' toxicity.

**Probiotic(s)**	**Probiotic dosage and treatment scheme**	**Therapeutic treatment scheme**	**Effect(s)**	**Reference**
*L. rhamnosus* GG	Daily intake of 1–2 × 10^10^ during the 24 weeks of adjuvant cancer chemotherapy	Monthly administration of 370–425 mg/m^2^ 5-FU repeated for six times or bimonthly administration of 400 mg/m^2^ as a bolus plus 48 h-infusion of 3.0–3.6 mg/m^2^ 5-FU, repeated for 12 times.	Decreased severe diarrhea, abdominal discomfort, and need to scale chemotherapy doses in colorectal cancer patients	(39)
*B. bifidum* G9-1	Daily administration of 10^7^-10^9^ CFU for 9 days, starting 3 days before the onset of chemotherapy	Daily injection of 50 mg 5-FU/kg body weight for 6 days	Milder intestinal mucositis and decreased weight loss in mice	(40)
*B. infantis*	Daily administration of 1 × 10^9^ CFU for 11 days, starting 7 days before chemotherapy	Single injection of 150 mg/kg body weight 5-FU	Decreased diarrhea occurrence, intestinal damage, and body weight loss in rats	(41)
*B. breve* DM8310 + *L. acidophilus* DM8302 + *L. casei* DM8121 + *S. thermophilus* DM8309	Daily administration of 1 × 10^9^ CFU/kg or 1 × 10^8^ CFU/kg for 8 days	Daily injection of 30 mg 5-FU/kg body weight for 5 consecutive days	Milder intestinal mucositis in rats	(42)
*L. casei* variety *rhamnosus* or *L. Acidophilus* + *B. Bifidum*	Daily administration of 1 × 10^7^ CFU for 5 days	Daily injection of 30 mg 5-FU/kg body weight for 5 days	Less severe intestinal damage, diarrhea, and body weight loss in mice	(43)
*B. breve* HA-129 + *B. bifidum* HA-132 + *B. longum* HA-135 + *L. rhamnosus* HA-111 + *L. acidophilus* HA-122 *+ L. casei* HA-108 + *L. plantarum* HA-119 + *S. thermophilus* HA-110 + *L. brevis* HA-112 + *B. infantis* HA-116	Daily intake of 30 × 10^9^ CFU for 12 weeks	Administration of irinotecan weekly or every 2–3 weeks	Decreased diarrhea incidence and severity, reduced enterocolitis, and bloating in colorectal cancer patients	(44)
*VSL#3*	Daily administration of 3 × 10^8^ CFU for 21 days before and days 7 after chemotherapy	Single dose of 225 mg irinotecan/kg body weight	Decreased weight, diarrhea severity, and intestinal damage	(45)
*VSL#3*	Three doses/day of 450 billions/g	Adjuvant post-operative radiotherapy. Total X-ray dose between 60 and 70 Gy	Decreased enterocolitis occurrence, incidence and severity of diarrhea, number of daily bowel movements, and delayed use of antidiarrheal drugs in sigmoid, rectal, or cervical cancer patients	(46)
	Bacteria during all the scheduled cycles of radiotherapy			
*L. acidophilus + B. bifidum*	Twice/day intake of 2 × 109 CFU beginning 7 days before and continuing everyday during radiotherapy	External pelvic radiotherapy with a dose 200 cGy per fraction, five fractions per week + weekly cisplatin 40 mg/m^2^ for 6 weeks	Decreased diarrhea severity and reduced use of anti-diarrheal drugs in cervical cancer patients	(47)
*L. acidophilus* LA-5 *+ B. animalis* subsp. *lactis* BB-12	Three times/daily intake of 1.75 billion bacteria for all the duration of radiotherapy	External beam pelvic radiotherapy with the standard dose of 50 Gy with or without concurrent chemotherapy, for 37 days	Decreased diarrhea incidence and severity, reduced abdominal pain, and use of anti-diarrheal drugs in cervical cancer patients	(48)
*L. brevis* CD2	Daily intake of 12 × 109 bacteria during and for one week after the end of therapy	Radical radiotherapy at a dose of 70 Gy in 35 fractions over 7 weeks (at 5 fractions per week) + weekly cisplatin 40 mg/m^2^ for 7 doses	Decreased incidence and severity of oral mucositis in head and neck cancer patients	(51)
*L. brevis* CD2	Daily intake of 12 × 109 bacteria	Intensity-modulated radiation therapy with ad dose fractionation of 68–70 Gy and 50–54 Gy to the macroscopic disease and low-risk regions, respectively + cisplatin using a weekly (40 mg/m^2^) or a 3-weekly (100 mg/m^2^) schedule.	No preventive effect on oral mucositis in head and neck cancer patients	(50)
*B. longum+ L. lactis + E. faecium*	Three capsules three times/day during all the treatmnet for up to 7 weeks	Intensity-modulated 70 Gy of radiotherapy in 32 fractions (2.19 Gy/day, 5 day/week), with the gross tumor volume receiving 60 Gy in 32 fractions for 45 days + 3 doses of 100 mg/m^2^ cisplatin every 3 weeks	Decreased severity of oral mucositis in nasopharyngeal carcinoma patients	(49)

## Probiotics as Vehicles for Drug Delivery and Gene Therapy

Another promising application of probiotics in the field of cancer treatment is represented by their use as vectors for drug delivery or gene therapy. Given the systemic toxicity of many chemo- and immunotherapeutic drugs, probiotic bacteria have been engineered to locally deliver the treatment at the tumor site (52–54) also by exploiting the affinity of anaerobic strains for the anaerobic tumor microenvironment (55). This has the double advantage of increasing drug efficacy and reducing adverse effects (52–54). In addition, probiotics strains have also been used to deliver anticancer proteins at the tumor site in order to inhibit its growth (55).

## Conclusion

**Figure 1:**
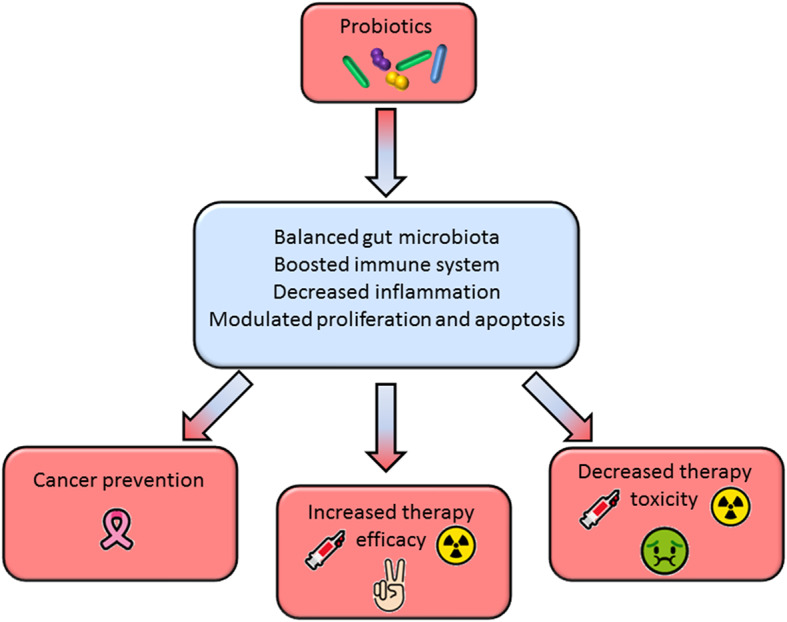
Beneficial effects of probiotics administration in cancer prevention and therapy.

A substantial body of literature in recent years has tried to shed light on the role of gut microbiota in the setting of cancer development and response to therapy. The studies reviewed in this paper strongly indicate that microbiota manipulation through selected probiotics may be a promising tool to prevent cancer onset, to improve clinical efficacy, and mitigate adverse effects of the standard anticancer therapies. Most of these benefits are achieved through the modulation of the host immunity and inflammatory response (Figure 1).

Overall, this review of the literature has demonstrated that comparable successful results can be provided by the use of single probiotics strains, as well as from different combinations thereof, although the dosage, frequency, and duration of both probiotic and drug administration may vary from study to study. In addition, it is evident that each probiotic strain or combination can prove effective in the setting of several cancers and of different chemotherapeutic protocols. However, the fact that a considerable number of reports come from experiments on preclinical models, may raise the need to further investigate the translatability of the animal findings to human patients.

Future clinical investigations on this topic are strongly required, with the aim to set up innovative approaches capable to improve clinical outcome and/or cancer patients' quality of life.

## Author Contributions

All authors listed have made a substantial, direct and intellectual contribution to the work, and approved it for publication.

## Conflict of Interest

The authors declare that the research was conducted in the absence of any commercial or financial relationships that could be construed as a potential conflict of interest.

## Footnotes

^1^https://www.who.int/news-room/fact-sheets/detail/cancer
